# Clonal Expansion of Multidrug-Resistant and Extensively Drug-Resistant Tuberculosis, Japan

**DOI:** 10.3201/eid1606.091844

**Published:** 2010-06

**Authors:** Yoshiro Murase, Shinji Maeda, Hiroyuki Yamada, Akihiro Ohkado, Kinuyo Chikamatsu, Kazue Mizuno, Seiya Kato, Satoshi Mitarai

**Affiliations:** The Research Institute of Tuberculosis/Japan Anti-Tuberculosis Association, Tokyo, Japan

**Keywords:** Mycobacterium tuberculosis, molecular epidemiology, MDR TB, XDR TB, RFLP, VNTR, spoligotyping, bacteria, tuberculosis and other mycobacteria, research

## Abstract

The emergence and spread of multidrug-resistant (MDR) and extensively drug-resistant (XDR) tuberculosis (TB) has raised public health concern about global control of TB. To estimate the transmission dynamics of MDR and XDR TB, we conducted a DNA fingerprinting analysis of 55 MDR/XDR *Mycobacterium tuberculosis* strains isolated from TB patients throughout Japan in 2002. Twenty-one (38%) of the strains were classified into 9 clusters with geographic links, which suggests that community transmission of MDR/XDR TB is ongoing. Furthermore, the XDR *M. tuberculosis* strains were more likely than the non–XDR MDR strains to be clustered (71% vs. 24%; p = 0.003), suggesting that transmission plays a critical role in the new incidence of XDR TB. These findings highlight the difficulty of preventing community transmission of XDR TB by conventional TB control programs and indicate an urgent need for a more appropriate strategy to contain highly developed drug-resistant TB.

## Abstract 2

Strain clustering suggests community transmission plays a critical role in incidence.

The epidemic of drug-resistant tuberculosis (TB) has raised public health concern about the global control of TB. The World Health Organization estimated that 0.5 million cases of multidrug-resistant TB (MDR TB) (i.e., *Mycobacterium tuberculosis* resistant to >2 of the most potent TB drugs, rifampin and isoniazid) occurred in 2007 (*1*). Some countries have extraordinarily high rates of this disease, but the problem is universal, and the extent varies from 1 country to another.

Another recent alarming issue is the emergence of extensively drug-resistant TB (XDR TB) (i.e., *M. tuberculosis* with MDR plus resistance to any fluoroquinolone and >1 injectable drug, thus posing even greater management challenges than MDR TB alone). The treatment outcome of XDR TB is worse than that of simple MDR TB, and the death rate is particularly high among HIV-infected patients (*2*). Also, because XDR TB is much more expensive to manage in terms of prolonged medication and prolonged period of infectivity to other persons (*3*), it has the potential to exhaust human and financial resources of the public health system for TB control. Although this new life-threatening disease had been reported from 49 countries as of June 2008 (*4*), its transmissibility among immunocompetent persons is not well known (*5*).

In Japan, TB remains a major infectious disease; in 2008, a total of 19.4 cases/100,000 population were reported (*6*), and Japan is generally classified as a country with intermediate TB incidence. According to the most recent nationwide drug-resistance survey, the prevalence of MDR TB and XDR TB were 1.9% and 0.5%, respectively (*7*). Approximately one third of MDR and XDR (MDR/XDR) *M. tuberculosis* strains were isolated from new TB patients, implying ongoing transmission of MDR/XDR TB in Japan.

Our purpose was to evaluate the transmission dynamics of MDR/XDR TB by using strains from the most recent (2002) nationwide drug-resistance survey in Japan, an industrialized country with low HIV incidence and intermediate TB incidence. We did so by analyzing the MDR/XDR strains by molecular genotyping methods, i.e., insertion sequence *6110* restriction fragment length polymorphism (IS*6110*-RFLP), spacer-oligonucleotide genotyping (spoligotyping), and variable number tandem repeats (VNTR).

## Materials and Methods

We used data and culture isolates obtained in the 2002 nationwide drug-resistance survey, as previously reported (*7*). Briefly, during June–November 2002, a total of 3,122 clinical strains were collected from different patients who had started treatment in 99 hospitals throughout Japan. The number of patients enrolled represented 36.0% of all new reported TB cases during the study period. The sampling of the hospital was not randomized but was based on voluntary participation. The survey identified 60 MDR/XDR *M. tuberculosis* strains, 55 of which were analyzed in this study; the other 5 strains were unavailable for use in this study.

### Patient Information

We used patient information collected from a standard data collection form in the drug-resistance survey in 2002 (*7*). The information included demographic data (age, sex, nationality, hospital, geographic area of the hospital), clinical data (history of TB treatment, site of TB disease, chest radiograph findings, underlying disease), and bacteriologic data (results of sputum smear test for acid-fast bacilli). Patients were classified as new if they had never been treated for TB for >4 weeks and as previously treated if they had ever been treated for TB for >4 weeks. The survey protocol conformed to the national guidelines for epidemiologic research (*8*).

### Drug Susceptibility Testing

Drug susceptibility testing was performed at the Research Institute of Tuberculosis, Tokyo, by using the proportion method on standard 1% Ogawa egg-based slants (*7*) and the following drug concentrations: isoniazid 0.2 µg/mL, rifampin 40 µg/mL, streptomycin 10 µg/mL, ethambutol 2.5 µg/mL, ethionamide 20 µg/mL, kanamycin 20 µg/mL, cycloserine 30 µg/mL, *p*-aminosalicylic acid 0.5 µg/mL, and levofloxacin 1 µg/mL. Pyrazinamide susceptibility was tested by using MGIT AST (Becton Dickinson, Sparks, MD, USA) at a concentration of 100 µg/mL. All compounds were obtained from Sigma (St. Louis, MO, USA).

### Definition of XDR TB

We defined XDR strains according to the World Health Organization definition of XDR (*1*) and drug availability for TB treatment in Japan. XDR strains were defined as *M. tuberculosis* strains with resistance to at least isoniazid, rifampin, levofloxacin, and kanamycin.

### Molecular Genotyping

Three molecular genotyping methods based on IS*6110*-RFLP, spoligotyping, and VNTR were performed on the 55 MDR/XDR strains. IS*6110*-RFLP typing was performed according to the standardized protocol (*9*). The RFLP band patterns were compared by using the BioNumerics software package version 5.1 (Applied Maths, Kortrijk, Belgium). Strains with an identical band pattern were classified as an RFLP cluster. Spoligotyping was also performed according to the standard protocol (*10*). Classification of the spoligotype family was performed according to the international database, SpolDB4 (*11*). The VNTR analysis was conducted as described elsewhere (*12*) by using the standard 15 mycobacterial interspersed repetitive unit–VNTR proposed by Supply et al. (*13*), i.e., VNTRs 0424, 0577, 0580, 0802, 0960, 1644, 1955, 2163b, 2165, 2401, 2996, 3192, 3690, 4052, and 4156 plus 4 other loci, VNTRs 2074, 2372, 3155, and 3336. The latter 4 loci were added to increase discrimination for the Beijing family strains because of their high prevalence in Japan (*12*).

### Statistical Analysis

Statistical analysis was performed with Epi Info software 3.51 (Centers for Disease Control and Prevention, Atlanta, GA, USA), by using χ^2^ test or Fisher exact test for the comparison of proportions. A p value <0.05 was considered significant.

## Results

### Epidemiologic Characteristics of Patients

A total of 55 MDR/XDR cases were analyzed. The characteristics of patients with MDR/XDR TB are summarized and compared with those of patients with pansusceptible strains (n = 2,782) in Table 1. Patients with MDR/XDR TB were significantly more likely to be younger (odds ratio [OR] 5.69 for age 21–40 years; 4.11 for age 41–60 years) and foreigners (OR 6.41) and to have been previously treated (OR 10.55). All MDR/XDR TB patients had pulmonary disease, and these patients were significantly more likely to have cavitary lesions (OR 3.24) and to have positive sputum smear test results (OR 2.20).

**Table 1:** Comparison of characteristics between TB patients with XDR TB and non-XDR MDR TB from the most recent (2002) nationwide drug susceptibility survey, Japan*

Characteristic	No. (%) cases					Odds ratio (95% confidence interval)	
	XDR, n = 17	Non–XDR MDR, n = 38	MDR/XDR, n = 55	Drug susceptible, n = 2,782		MDR/XDR vs. drug susceptible	XDR vs. non–XDR MDR
Age, y							
0–20	0	1 (3)	1 (2)	51 (2)		2.57 (–)	–
21–40	6 (35)	14 (37)	20 (36)	460 (17)		**5.69 (2.63–12.45)**	0.51 (0.12–2.18)
41–60	10 (59)	12 (32)	22 (40)	701 (25)		**4.11 (1.93–8.85)**	1
>61	1 (6)	11 (29)	12 (22)	1,570 (56)		1	0.11 (0.00–1.11)
Sex							
M	9 (53)	30 (79)	39 (71)	1,973 (71)		1	1
F	8 (47)	8 (21)	16 (29)	809 (29)		1.00 (0.53–1.86)	3.33 (0.83–13.77)
Nationality							
Japanese	16 (94)	31 (82)	47 (85)	2,710 (97)		1	1
Foreigner	1 (6)	7 (18)	8 (15)	72 (3)		**6.41 (2.69–14.72)**	0.28 (0.01–2.64)
Treatment history							
New	8 (47)	17 (45)	25 (45)	2,498 (90)		1	1
Previous	9 (53)	21 (55)	30 (55)	284 (10)		**10.55 (5.93–18.83)**	0.91 (0.25–3.33)
Site of TB							
Pulmonary	17 (100)	38 (100)	55 (100)	2,687 (97)		–	–
Extrapulmonary	0	0	0	95 (3)		–	–
Chest radiograph finding							
Noncavitary	5 (29)	8 (21)	13 (24)	1,394 (50)		1	1
Cavitary	12 (71)	30 (79)	42 (76)	1,388 (50)		**3.24 (1.68–6.38)**	0.64 (0.15–2.84)
Sputum smear test result							
Negative	3 (18)	6 (16)	9 (16)	838 (30)		1	1
Positive	14 (82)	32 (84)	46 (84)	1,944 (70)		**2.20 (1.03–4.85)**	0.88 (0.16–5.23)
Complication							
None	6 (35)	21 (55)	27 (49)	1,344 (48)		1	
Diabetes mellitus	4 (24)	7 (18)	11 (20)	438 (16)		1.25 (0.58–2.65)	2.00 (0.34–11.88)
Malignancy	3 (18)	2 (5)	5 (9)	180 (6)		1.38 (0.46–3.83)	5.25 (0.52–61.86)

*TB, tuberculosis; XDR, extensively drug-resistant; MDR, multidrug-resistant; MDR/XDR, MDR TB and XDR TB; –, not available. **Boldface** indicates significance.

Of the 55 MDR/XDR TB cases, 17 (31%) were XDR TB. We found no significant differences between patients with XDR TB and patients with MDR but not XDR (non–XDR MDR) TB. XDR TB patients tended to be female, although the difference was not statistically significant (p = 0.06, Fisher exact test).

### Analysis by Spoligotyping

We performed spoligotyping to determine the population structure of the 55 MDR/XDR strains (Table 2). The most dominant spoligotype family in the MDR/XDR cases was the Beijing family genotype (62%, n = 34), followed by the T (13%, n = 7), Latino-American and Mediterranean (5%, n = 3), U (2%, n = 1), East-African Indian (2%, n = 1), and X (2%, n = 1) family genotypes. Eight (15%) strains were unclassified according to the database.

**Table 2:** Distribution of *Mycobacterium tuberculosis* spoligotype families among 55 persons with MDR/XDR TB, Japan*

Spoligotype family	No. (%) cases		
	XDR TB, n = 17	Non-XDR MDR TB, n = 38	MDR and XDR TB, n = 55
Beijing†	8 (47)	26 (68)	34 (62)
T	2 (12)	5 (13)	7 (13)
LAM	3 (18)	0	3 (5)
U	0	1 (3)	1 (2)
EAI	0	1 (3)	1 (2)
X	0	1 (3)	1 (2)
Unclassified‡	4 (24)	4 (11)	8 (15)

*MDR, multidrug-resistant; XDR, extensively drug-resistant; TB, tuberculosis; LAM, Latino-American and Mediterranean; EAI, East-African Indian. †Includes Beijing-like strains. ‡Unclassified according to the SpolDB4.

The proportion of the Beijing family, which is frequently reported to be associated with drug resistance (*14*), did not significantly differ between the non–XDR MDR *M. tuberculosis* strains and XDR *M. tuberculosis* strains (68% vs. 47%; p = 0.14), and distribution of the other spoligotype families did not differ significantly (data not shown).

### Cluster Analysis by IS*6110*-RFLP

All 55 MDR/XDR strains were genotyped by IS*6110*-RFLP, and 21 (38%) were classified into 9 clusters, each with identical RFLP patterns (clusters 1–9) (Appendix Figure). All members of each cluster belonged to the same spoligotype family. The remaining 34 strains exhibited unique RFLP patterns. Cluster size ranged from 2 to 4, and 7 clusters had 2 members, 1 with 3 members and 1 with 4 members. IS*6110* copy numbers ranged from 1 to 18. Four (7%) strains had <5 copies of IS*6110*, and these strains were discriminated as unique strains by the subsequent VNTR analysis. Of the 21 RFLP-clustered strains, 13 (62%) were classified as XDR *M. tuberculosis*, and 8 (38%) were isolated from new TB patients. Of the 17 XDR *M. tuberculosis* strains, 4 were resistant to all drugs tested, and 2 belonged to cluster 8. Among the 9 RFLP-clusters, 7 were from Japanese patients exclusively; the other 2 clusters were from both Japanese and foreign-born patients.

### Cluster Analysis by VNTR

The results of the 19-locus VNTR (15 mycobacterial interspersed repetitive unit–VNTR + additional 4 loci) analysis showed that 7 of the 9 RFLP-clusters were identical according to the 19-locus VNTR (Appendix Figure). A difference in only 1 locus was observed in the remaining 2 RFLP-clusters: 1 at VNTR1955 in cluster 6, and 1 at VNTR2163b in cluster 9 (Appendix Figure).

### Geographic Distribution of Hospitals with MDR/XDR TB Patients among Each Cluster

To estimate the possible clonal expansion of MDR/XDR TB in communities, we compared the geographic areas of patients’ hospitals among each cluster (Table 3). The 55 MDR/XDR TB patients were treated by 23 hospitals, which were located in 16 of the 47 prefectures in Japan. Of the 9 clusters, 7 consisted of patients whose hospitals were located in the same or neighboring prefectures, and the remaining 2 clusters consisted of patients whose hospitals were located in the same region (i.e., a geographic and administrative area with several prefectures). Clusters with geographic links suggest the clonal expansion of MDR/XDR TB occurred in local areas. Although patients in clusters 4 and 7 were from the same hospital, further information about their contact was unavailable because an epidemiologic survey was not performed.

**Table 3:** Geographic distribution of hospitals among each cluster of MDR and XDR TB, Japan*

Cluster no.	Patient ID	Type of TB	Hospital	Geographic link of the hospitals in clusters
1	DR43	MDR	A	
	DR42	XDR	B	A and B located in same prefecture
2	DR14	MDR	C	
	DR53	MDR	D	C located 103 km from D
3	DR54	XDR	D	
	DR58	XDR	D	
	DR18	XDR	E	D and E located in same prefecture
4	DR03	MDR	F	
	DR04	MDR	F	Same hospital
5	DR11	MDR	G	
	DR10	MDR	H	G located 221 km from H
6	DR51	XDR	D	
	DR50	XDR	D	
	DR55	MDR	D	
	DR16	XDR	E	D and E located in same prefecture
7	DR38	XDR	D	
	DR39	MDR	D	Same hospital
8	DR13	XDR	I	
	DR56	XDR	D	I and D located in neighboring prefectures
9	DR12	XDR	I	
	DR49	XDR	D	I and D located in neighboring prefectures

*MDR, multidrug-resistant; TB, tuberculosis; XDR, extensively drug-resistant.

### Characteristics of Clustered Cases

We analyzed the associations of genetic clustering by IS*6110*-RFLP with patients’ characteristics and bacteriologic factors (Table 4). XDR TB occurred more frequently among the clustered cases than among the unique cases (OR 7.73), but differences between the 2 groups were not significant for any of the remaining factors i.e., age, sex, nationality, treatment history, site of TB disease, chest radiographic findings, sputum-smear test results, underlying disease, or member of the Beijing family genotype.

**Table 4:** Comparison of MDR/XDR TB patients and bacteriologic characteristics between clustered and nonclustered cases by IS*6110*-RFLP, Japan*

Characteristic	No. (%) cases			Odds ratio (95% confidence interval)
	Clustered, n = 21	Unique, n = 34		Clustered vs. unique
Age, y				
0–20	1 (5)	0		–
21–40	8 (38)	12 (35)		0.96 (0.23–3.95)
41–60	9 (43)	13 (38)		1
>61	3 (14)	9 (26)		0.48 (0.08–2.83)
Sex				
M	12 (57)	27 (79)		1
F	9 (43)	7 (21)		2.89 (0.75–11.45)
Nationality				
Japanese	19 (90)	28 (82)		1
Foreigner	2 (10)	6 (18)		0.49 (0.06–3.18)
Treatment history				
New	8 (38)	8 (24)		1
Previous	13 (62)	26 (76)		0.50 (0.13–1.90)
Site of TB				
Pulmonary	21 (100)	34 (100)		–
Extrapulmonary	0	0		–
Chest radiograph finding				
Noncavitary	6 (29)	7 (21)		1
Cavitary	15 (71)	27 (79)		0.65 (0.15–2.71)
Sputum smear test result				
Negative	4 (19)	5 (15)		1
Positive	17 (81)	29 (85)		0.73 (0.14–3.86)
Complication				
None	9 (43)	18 (53)		1
Diabetes mellitus	5 (24)	6 (18)		1.67 (0.32–8.78)
Malignancy	2 (10)	3 (9)		1.33 (0.13–12.94)
XDR TB				
No	9 (43)	29 (85)		1
Yes	12 (57)	5 (15)		**7.73 (1.84**–**34.83)**
Beijing family genotype				
No	6 (29)	15 (44)		1
Yes	15 (71)	19 (56)		1.97 (0.57–7.46)

*MDR, multidrug-resistant; XDR, extensively drug-resistant; TB, tuberculosis; IS*6110*-RFLP, insertion sequence *6110* restriction fragment length polymorphism –, not available. **Boldface** indicates significance.

## Discussion

Our study described the transmission dynamics of MDR/XDR TB in Japan on a national scale. Analysis of the 55 MDR/XDR TB cases showed that MDR/XDR TB was more frequent among younger patients, previously treated patients, and foreign-born patients than among patients with drug-susceptible TB (Table 1). In addition, the XDR TB cases, which represented 31% of the total MDR TB cases, were more likely to be associated with clustering than were the non–XDR MDR TB cases (Table 4), suggesting that ongoing transmission plays a critical role in new cases of XDR TB. We also found that the Beijing family genotype predominated in MDR/XDR *M. tuberculosis* (Table 2) and in drug-susceptible *M. tuberculosis* in this setting (*12*).

Although IS*6110*-RFLP analysis has been the standard for strain typing in studies of *M. tuberculosis* transmission, false clusters comprising strains without any epidemiologic link, and thus not reflecting clonal transmission, have been reported (*15*–*17*). This use of IS*6110*-RFLP analysis is especially problematic in area in which strains with stable RFLP patterns are endemic (*18*,*19*). In this context, because of its post hoc nature, a limitation of our study is its lack of information about epidemiologic links among clustered patients.

We therefore evaluated the genetic clonality of RFLP-clustered strains more rigorously by using the 19-locus VNTR. Our previous study demonstrated that most of the RFLP-clustered strains without epidemiologic links were discriminated by the 19-locus VNTR (*12*). In this study, the results correlated strongly between RFLP and VNTR in terms of cluster formation; 7 of the 9 RFLP-clusters had completely identical 19-locus VNTR profiles (Appendix Figure). Each of the remaining 2 clusters contained a single-locus variant, i.e., the difference was at only 1 locus of 1 strain in each cluster. This minor difference in VNTR profile is highly likely to have occurred as a random variation in strains from the same source, as argued by several investigators (*13*,*20*). In addition to analyzing by RFLP and VNTR, we confirmed that all of the 9 clusters shared identical mutations on drug resistance genes for rifampin and isoniazid (i.e., *rpoB* and *katG*, respectively, data not shown).

Geographic distribution of the hospitals also supports the clonal expansion of MDR/XDR TB (Table 3). Isolation of most of the clustered strains from hospitals in the same or neighboring prefectures may indicate that transmission is occurring in the communities. Furthermore, we assume that some of the clustering in our study did not result from direct transmission among the members but rather resulted from exposure of the members to a common infection source or infection with different sources sharing a near ancestor. The discordance of drug resistance other than isoniazid and rifampin resistance among clusters implies that the stepwise acquisition of drug resistance had occurred chronologically during successive transmissions (Appendix Figure). Thus, all these findings support the assumption of ongoing community transmissions of MDR/XDR TB.

A high proportion (71%, 12/17) of the XDR strains were involved in clusters, a finding consistent with the results of a hospital-based study in Osaka, Japan (*21*). Of the 12 clustered cases, 4 were new cases and 8 were among previously treated patients. The clustered XDR TB cases among the new cases strongly indicated that these persons had been primarily infected with XDR *M. tuberculosis* strains. Also, TB may have developed in the clustered XDR TB patients with previous TB treatment as a result of exogenous reinfection with XDR TB, as described in a report of fatal TB in a patient infected with an MDR strain when his disease had been almost cured (*22*).

One possible explanation for the high clustering rate of XDR TB is that new cases of XDR TB are more likely to be caused by transmission than by acquisition of multiple drug resistance as a result of treatment failure. Shah et al. reported that patients with XDR TB were more likely than those without XDR TB to be infectious in terms of duration and proportion of sputum smear positivity (*3*). Furthermore, at least 142 non–XDR MDR TB and 180 XDR TB patients were reported to be in Japan as of 2000 (*23*). All were culture positive, and a considerable number were treated as outpatients despite their infectiousness and drug resistance. Thus, we assume that some of those patients with chronic MDR/XDR TB may have played a role as a source of transmission, as described in this study.

Because these findings and our cluster analysis suggest that the current TB control strategy is insufficient to prevent community transmission of MDR/XDR TB, more detailed investigations of MDR/XDR TB transmission based on contact tracing are urgently needed to improve the infection control strategy, including an isolation policy for highly infectious patients with refractory drug-resistant strains. At the same time, ethical issues, such as the human rights of these patients, should be carefully considered.

Only a few cases of XDR TB transmission have ever been reported. A large group of XDR TB cases, mainly among HIV-infected patients, was reported in South Africa (*2*). Another study in South Africa reported 12 cases of exogenous reinfection with XDR TB (*24*). In Iran, DNA fingerprinting analysis suggested 2 outbreaks of XDR TB involving 12 patients in 1 family and their close contacts (*25*). In Norway, a patient who was lost to follow-up has been transmitting an XDR *M. tuberculosis* strain for 12 years, and that transmission has ultimately resulted in 15 XDR TB cases (*26*). Consistent with these previous reports, our study has added novel evidence for clonal expansion of XDR *M. tuberculosis* strains even in an industrialized country with intermediate TB incidence and low incidence of HIV.

Another limitation of this study is a possible sampling bias, which could be caused by the following factors. First, the sampling of the survey participants was not randomized but was based on voluntary participation of the hospitals, which may be likely to include more serious TB cases (*7*). Second, the sampling fraction is low (38%) and the study period is short (6 months), either of which may produce a biased subset of the total cases. In addition, the low sampling fraction and the short study period may lead to reduced clustering of cases (*27*,*28*). A more complete understanding of transmission dynamics of MDR/XDR TB requires a real-time DNA fingerprinting method such as VNTR on a nationwide scale.

Four strains from the 55 MDR/XDR TB cases were identified as totally drug-resistant strains (*29*), indicating that they were resistant to all 10 drugs tested (Appendix Figure). Of these strains, 2 in 1 cluster (cluster 8) were from new cases, and both patients were women in their 20s who were full-time workers and had no underlying disease. Both patients were registered in the same area (no further data available); TB as formidable as totally drug-resistant TB can affect otherwise healthy young adults even on a mass basis.

The results of this study showed that transmission of MDR TB, especially XDR TB, is currently occurring in communities of Japan. Further studies to prospectively identify the transmission route through contact tracing and real-time DNA fingerprinting methods on a population basis are required. Although the MDR/XDR TB problem is not great in Japan, our findings highlight the relevance of proper infection control, as well as effective treatment, to further contain highly developed drug-resistant TB.

## Supplementary Material

Appendix Figure Insertion sequence 6110 restriction fragment length polymorphism (IS6110-RFLP) patterns, copy number profiles of the 19-locus variable number tandem repeat (VNTR) profiles, spoligotype family, drug-resistance profiles, comparison of extensively drug-resistant (XDR) tuberculosis (TB) and multidrug-resistant but not XDR TB (non-XDR) cases, treatment (treat.) history, nationality (nat.), and sex. The dendrogram of the 55 MDR/XDR Mycobacterium tuberculosis strains was created on the basis of their IS6110-RFLP patterns. Seventeen strains were grouped into 9 RFLP clusters (gray), and the members of each group belonged to same spoligotype (spoil.) families. Of the 9 RFLP-clusters, 7 clusters had identical 19-locus VNTR (15 mycobacterial interspersed repetitive unit [MIRU]-VNTR + additional 4 loci) profiles, and the remaining 2 clusters each contained a single-locus variant (boxed in cluster 6 and cluster 9). Of the 55 MDR/XDR M. tuberculosis strains, 17 (31%) were classified as XDR strains, and 25 strains (45%) were isolated from new TB patients. LAM, Latino-American Mediterranean; EAI, East-African Indian; H, isoniazid; R, rifampin; Z, pyrazinamide; E, ethambutol, S, streptomycin; L, levofloxacin; K, kanamycin; T, ethionamide; C, cycloserine; P, p-aminosalicylic acid; Jpn, Japanese; non-Jpn, non-Japanese.
