# Supplementary figures and images for: Clonal Expansion of Multidrug-Resistant and Extensively Drug-Resistant Tuberculosis, Japan
Source: Emerg Infect Dis. 2010 Jun;16(6):948–54. doi: 10.3201/eid1606.091844 (PMC3086214; doi:10.3201/eid1606.091844)

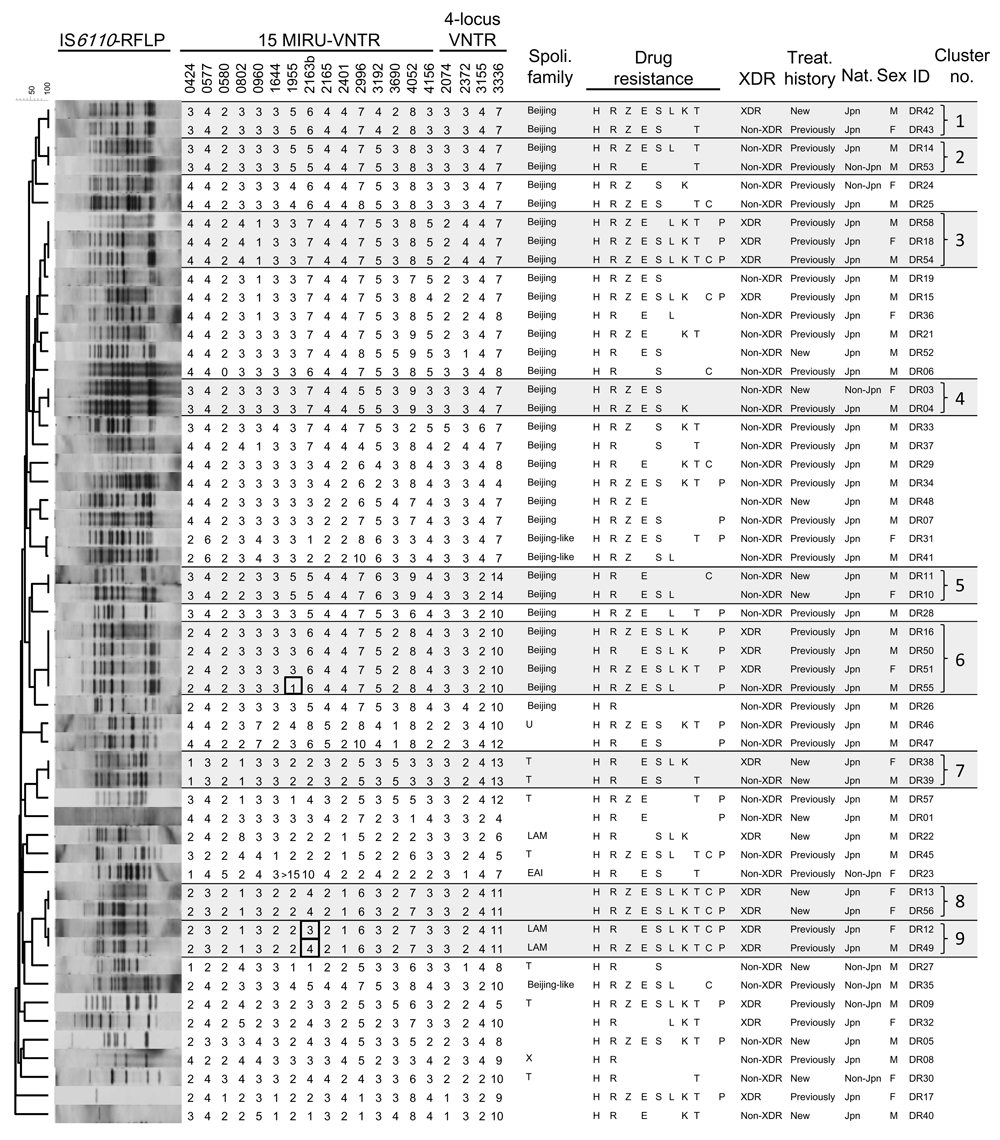

Supplement: Appendix Figure — Insertion sequence 6110 restriction fragment length polymorphism (IS6110-RFLP) patterns, copy number profiles of the 19-locus variable number tandem repeat (VNTR) profiles, spoligotype family, drug-resistance profiles, comparison of extensively drug-resistant (XDR) tuberculosis (TB) and multidrug-resistant but not XDR TB (non-XDR) cases, treatment (treat.) history, nationality (nat.), and sex. The dendrogram of the 55 MDR/XDR Mycobacterium tuberculosis strains was created on the basis of their IS6110-RFLP patterns. Seventeen strains were grouped into 9 RFLP clusters (gray), and the members of each group belonged to same spoligotype (spoil.) families. Of the 9 RFLP-clusters, 7 clusters had identical 19-locus VNTR (15 mycobacterial interspersed repetitive unit [MIRU]-VNTR + additional 4 loci) profiles, and the remaining 2 clusters each contained a single-locus variant (boxed in cluster 6 and cluster 9). Of the 55 MDR/XDR M. tuberculosis strains, 17 (31%) were classified as XDR strains, and 25 strains (45%) were isolated from new TB patients. LAM, Latino-American Mediterranean; EAI, East-African Indian; H, isoniazid; R, rifampin; Z, pyrazinamide; E, ethambutol, S, streptomycin; L, levofloxacin; K, kanamycin; T, ethionamide; C, cycloserine; P, p-aminosalicylic acid; Jpn, Japanese; non-Jpn, non-Japanese. [file 09-1844-appF-s1.gif]
